# Supplementary material for: Characterization of the bacterial microbiota composition and evolution at different intestinal tract in wild pigs (Sus scrofa ussuricus)
Source: PeerJ. 2020 May 26;8:e9124. doi: 10.7717/peerj.9124 (PMC7258971; doi:10.7717/peerj.9124)
Supplement: Table S1 [file peerj-08-9124-s001.docx]

**Table S1:**

**Overview of high-throughput sequencing data in different gut location samples from wild pigs**

| Sample | Raw PE | Effectives Tags |
| --- | --- | --- |
| DU1 | 57878 | 38980 |
| DU2 | 55796 | 40974 |
| DU3 | 59870 | 48502 |
| JE1 | 64106 | 51485 |
| JE2 | 58919 | 48631 |
| JE3 | 69602 | 51436 |
| IL1 | 55630 | 42654 |
| IL2 | 56416 | 50672 |
| IL3 | 58260 | 49158 |
| CE1 | 63192 | 41419 |
| CE2 | 56351 | 40214 |
| CE3 | 68051 | 46327 |
| CO1 | 72466 | 49125 |
| CO2 | 67312 | 44296 |
| CO3 | 61444 | 43604 |
| Total sequence | 92529 | 687477 |
