# Supplementary material for: Characterization of the bacterial microbiota composition and evolution at different intestinal tract in wild pigs (Sus scrofa ussuricus)
Source: PeerJ. 2020 May 26;8:e9124. doi: 10.7717/peerj.9124 (PMC7258971; doi:10.7717/peerj.9124)
Supplement: Table S3 [file peerj-08-9124-s003.docx]

**Table S3:**

**OTU numbers of the different gut intestinal samples at different taxonomy levels**

| Sample | Phylum | Class | Order | Family | Genus | Species | Unclassified |
| --- | --- | --- | --- | --- | --- | --- | --- |
| DU1 | 1906 | 1906 | 1897 | 1768 | 1278 | 512 | 0 |
| DU2 | 1837 | 1837 | 1833 | 1742 | 1219 | 460 | 3 |
| DU3 | 1969 | 1969 | 1960 | 1829 | 1118 | 346 | 0 |
| JE1 | 1310 | 1310 | 1304 | 1260 | 995 | 490 | 0 |
| JE2 | 1261 | 1261 | 1261 | 1229 | 943 | 434 | 0 |
| JE3 | 1572 | 1572 | 1568 | 1512 | 927 | 276 | 0 |
| IL1 | 1292 | 1292 | 1290 | 1263 | 955 | 386 | 0 |
| IL2 | 1449 | 1449 | 1443 | 1337 | 788 | 139 | 4 |
| IL3 | 1392 | 1392 | 1388 | 1342 | 866 | 198 | 0 |
| CE1 | 1815 | 1815 | 1813 | 1568 | 878 | 391 | 0 |
| CE2 | 1820 | 1820 | 1817 | 1564 | 947 | 427 | 0 |
| CE3 | 2231 | 2231 | 2218 | 1813 | 615 | 75 | 0 |
| CO1 | 1913 | 1913 | 1911 | 1666 | 1049 | 496 | 0 |
| CO2 | 2279 | 2279 | 2274 | 1967 | 956 | 335 | 0 |
| CO3 | 1480 | 1480 | 1477 | 1259 | 721 | 234 | 0 |
