# Supplementary material for: Characterization of the bacterial microbiota composition and evolution at different intestinal tract in wild pigs (Sus scrofa ussuricus)
Source: PeerJ. 2020 May 26;8:e9124. doi: 10.7717/peerj.9124 (PMC7258971; doi:10.7717/peerj.9124)
Supplement: Table S4 [file peerj-08-9124-s004.docx]

**Table S4:**

**Microbial composition of the five gut intestinal in wild pigs at the phylum level.**

| Taxon | Duodenum | Jejunum | Ileum | Cecum | Colon |
| --- | --- | --- | --- | --- | --- |
| Firmicutes | 0.3884 | 0.3222 | 0.3045 | 0.5235 | 0.4545 |
| Actinobacteria | 0.4354 | 0.4666 | 0.3122 | 0.3493 | 0.3441 |
| Proteobacteria | 0.0246 | 0.2044 | 0.3239 | 0.0110 | 0.0092 |
| Bacteroidetes | 0.1160 | 0.0040 | 0.0510 | 0.0973 | 0.1686 |
| Cyanobacteria | 0.0310 | 0.0019 | 0.0024 | 0.0010 | 0.0016 |
| Verrucomicrobia | 0.0018 | 0.0001 | 0.0020 | 0.0057 | 0.0135 |
| Spirochaetes | 0.0011 | 0.0000 | 0.0011 | 0.0103 | 0.0071 |
| TM7 | 0.0006 | 0.0006 | 0.0005 | 0.0009 | 0.0010 |
| Tenericutes | 0.0001 | 0.0000 | 0.0001 | 0.0005 | 0.0002 |
| Fibrobacteres | 0.0000 | 0.0000 | 0.0000 | 0.0004 | 0.0002 |
| Synergistetes | 0.0002 | 0.0001 | 0.0002 | 0.0000 | 0.0001 |
| Lentisphaerae | 0.0000 | 0.0000 | 0.0005 | 0.0000 | 0.0000 |
| [Thermi] | 0.0000 | 0.0000 | 0.0003 | 0.0000 | 0.0000 |
| Planctomycetes | 0.0000 | 0.0001 | 0.0001 | 0.0000 | 0.0000 |
| Armatimonadetes | 0.0000 | 0.0000 | 0.0001 | 0.0000 | 0.0000 |
| OD1 | 0.0000 | 0.0000 | 0.0001 | 0.0000 | 0.0000 |
| Acidobacteria | 0.0000 | 0.0000 | 0.0001 | 0.0000 | 0.0000 |
