# Supplementary material for: Characterization of the bacterial microbiota composition and evolution at different intestinal tract in wild pigs (Sus scrofa ussuricus)
Source: PeerJ. 2020 May 26;8:e9124. doi: 10.7717/peerj.9124 (PMC7258971; doi:10.7717/peerj.9124)
Supplement: Table S5 [file peerj-08-9124-s005.docx]

**Table S5:**

**Microbial composition of the five gut intestinal in wild pigs at the genus level.**

| Taxon | Duodenum | Jejunum | Ileum | Cecum | Colon |
| --- | --- | --- | --- | --- | --- |
| Bifidobacterium | 0.3487 | 0.3110 | 0.1721 | 0.2248 | 0.2740 |
| Unclassified_Coriobacteriaceae | 0.0651 | 0.1352 | 0.1093 | 0.1212 | 0.0680 |
| Allobaculum | 0.0208 | 0.0473 | 0.0426 | 0.1497 | 0.1280 |
| Unclassified_Clostridiaceae | 0.0762 | 0.0239 | 0.0558 | 0.1190 | 0.0521 |
| Psychrobacter | 0.0036 | 0.1239 | 0.1750 | 0.0000 | 0.0006 |
| Lactobacillus | 0.1163 | 0.1105 | 0.0388 | 0.0036 | 0.0206 |
| Unclassified_Clostridiales | 0.0194 | 0.0368 | 0.0165 | 0.0591 | 0.0484 |
| Unclassified_Lachnospiraceae | 0.0263 | 0.0189 | 0.0199 | 0.0496 | 0.0480 |
| Prevotella | 0.0997 | 0.0024 | 0.0429 | 0.0056 | 0.0027 |
| Unclassified_Ruminococcaceae | 0.0023 | 0.0004 | 0.0016 | 0.0641 | 0.0517 |
| Unclassified_Moraxellaceae | 0.0054 | 0.0495 | 0.0598 | 0.0000 | 0.0001 |
| Wautersiella | 0.0000 | 0.0000 | 0.0000 | 0.0000 | 0.1115 |
| Unclassified_Bacteroidales | 0.0054 | 0.0000 | 0.0014 | 0.0628 | 0.0279 |
| Clostridium | 0.0209 | 0.0079 | 0.0204 | 0.0200 | 0.0100 |
| Megasphaera | 0.0404 | 0.0010 | 0.0312 | 0.0001 | 0.0000 |
| Sutterella | 0.0040 | 0.0024 | 0.0389 | 0.0105 | 0.0049 |
| Unclassified_[Mogibacteriaceae] | 0.0045 | 0.0214 | 0.0120 | 0.0024 | 0.0044 |
| Unclassified_Peptostreptococcaceae | 0.0073 | 0.0049 | 0.0033 | 0.0200 | 0.0090 |
| Bulleidia | 0.0096 | 0.0228 | 0.0096 | 0.0000 | 0.0002 |
| Solibacillus | 0.0000 | 0.0000 | 0.0000 | 0.0000 | 0.0394 |
| Unclassified_S24-7 | 0.0042 | 0.0000 | 0.0029 | 0.0132 | 0.0141 |
| Unclassified_Streptophyta | 0.0304 | 0.0013 | 0.0013 | 0.0000 | 0.0000 |
| Turicibacter | 0.0050 | 0.0071 | 0.0095 | 0.0016 | 0.0011 |
| Ochrobactrum | 0.0024 | 0.0040 | 0.0145 | 0.0000 | 0.0002 |
| Shewanella | 0.0000 | 0.0000 | 0.0207 | 0.0000 | 0.0000 |
| Pseudomonas | 0.0001 | 0.0188 | 0.0011 | 0.0000 | 0.0000 |
| Treponema | 0.0005 | 0.0000 | 0.0003 | 0.0100 | 0.0068 |
| Unclassified_Veillonellaceae | 0.0040 | 0.0001 | 0.0111 | 0.0001 | 0.0001 |
| Sharpea | 0.0023 | 0.0072 | 0.0045 | 0.0003 | 0.0012 |
| Parabacteroides | 0.0038 | 0.0010 | 0.0013 | 0.0049 | 0.0044 |
| Epulopiscium | 0.0075 | 0.0019 | 0.0024 | 0.0008 | 0.0021 |
| SMB53 | 0.0020 | 0.0013 | 0.0012 | 0.0066 | 0.0036 |
| Others | 0.0619 | 0.0371 | 0.0781 | 0.0501 | 0.0649 |
